# How to precisely measure the volume velocity transfer function of physical vocal tract models by external excitation

**DOI:** 10.1371/journal.pone.0193708

**Published:** 2018-03-15

**Authors:** Mario Fleischer, Alexander Mainka, Steffen Kürbis, Peter Birkholz

**Affiliations:** 1 Division of Phoniatrics and Audiology, Department of Otorhinolaryngology, Faculty of Medicine Carl Gustav Carus, Technische Universität Dresden, Fetscherstrasse 74, 01307 Dresden, Germany; 2 Voice Research Laboratory, Hochschule für Musik Carl Maria von Weber Dresden, Wettiner Platz 13, 01067 Dresden, Germany; 3 Institute of Acoustics and Speech Communication, Faculty of Electrical and Computer Engineering, Technische Universität Dresden, Helmholtzstrasse 18, 01062 Dresden, Germany; University of Sussex, UNITED KINGDOM

## Abstract

Recently, 3D printing has been increasingly used to create physical models of the vocal tract with geometries obtained from magnetic resonance imaging. These printed models allow measuring the vocal tract transfer function, which is not reliably possible in vivo for the vocal tract of living humans. The transfer functions enable the detailed examination of the acoustic effects of specific articulatory strategies in speaking and singing, and the validation of acoustic plane-wave models for realistic vocal tract geometries in articulatory speech synthesis. To measure the acoustic transfer function of 3D-printed models, two techniques have been described: (1) excitation of the models with a broadband sound source at the glottis and measurement of the sound pressure radiated from the lips, and (2) excitation of the models with an external source in front of the lips and measurement of the sound pressure inside the models at the glottal end. The former method is more frequently used and more intuitive due to its similarity to speech production. However, the latter method avoids the intricate problem of constructing a suitable broadband glottal source and is therefore more effective. It has been shown to yield a transfer function similar, but not exactly equal to the volume velocity transfer function between the glottis and the lips, which is usually used to characterize vocal tract acoustics. Here, we revisit this method and show both, theoretically and experimentally, how it can be extended to yield the precise volume velocity transfer function of the vocal tract.

## Introduction

The vocal tract transfer function, i.e., the complex frequency-dependent ratio of the volume velocity (or alternatively sound pressure) at the lips to the volume velocity through the glottis, is widely used to characterize the acoustics of the vocal tract. It contains the information about the frequencies and bandwidths of the formants (resonances), which are of primary importance in many studies. Besides the formants, most transfer functions contain additional information in terms of close pole-zero pairs, which are caused by side cavities like the piriform sinus, the vallecula, a cavity between tongue base and epiglottis, interdental spaces, or the nasal cavity [1–3]. The measurement of the complete transfer function of real vocal tract geometries with a bandwidth of up to at least 6 kHz (speech range) and a high signal-to-noise ratio is therefore of paramount interest.

The only known direct method to measure the vocal tract transfer function *in vivo* requires an external broadband excitation of the vocal tract with a transducer placed in the vicinity of the larynx, while measuring the sound pressure radiated from the mouth [4–6]. Because the sound of the source must pass the tissue of the neck to excite the air in the vocal tract, the source must be firmly pressed against the neck, which can be inconvenient. Furthermore, depending on the subject, the damping by the tissue may be so strong that the signal-to-noise ratio of the recorded signal is not high enough to be useful.

A more convenient direct method to determine the formant frequencies (but not the full volume velocity transfer function) excites the vocal tract with a volume velocity source close to the mouth opening and simultaneously measures its sound pressure response right next to the source [7–9]. The quotient of the recorded pressure and the emitted volume velocity is the impedance of the vocal tract in parallel with the radiation impedance, the peaks of which correspond to the radiation-loaded vocal tract resonances [7]. However, it is very difficult to construct a volume velocity source with a flat response over a sufficiently high bandwidth, and the radiation from the mouth is physically disturbed by the source and the microphone. Furthermore, the input impedance differs from the volume velocity transfer function that is usually used to characterize vocal tract acoustics.

A relatively new method to obtain a detailed vocal tract transfer function of a sustained speech sound consists of measuring the vocal tract shape using 3D magnetic resonance imaging (MRI), segmenting the vocal tract shape from the MRI data, printing a 1:1 physical model of the shape using a 3D printer, and measuring the transfer function of the physical model [2, 10–12]. The advantage in contrast to measurements in humans is that the printed models have no limitations with respect to the placement of sound sources and microphones. Two approaches have been described to obtain the transfer function of these models.

One approach is to excite the models with a broadband sound source at the glottis and measure the sound pressure radiated from the lips [11–13]. Here, the sound source should ideally be a volume velocity source with an infinite output impedance, which is hence independent of the vocal tract load as assumed by the source-filter theory of speech production [14]. However, constructing and calibrating a broadband volume velocity source with a flat frequency response is intricate. Some studies used a loudspeaker or horn driver connected to an impedance matching horn with a small (≤ 4 mm) annular aperture at the distal end of the horn, which is attached to the glottal end of the vocal tract model [13, 15]. Alternatively, the horn is omitted and the speaker is directly attached to a connector plate with a small hole [12]. For a good approximation of a volume velocity source, the hole must be so small that its acoustic resistance is much higher than the highest input impedance of the models. However, the high resistance of the hole and the cavity resonances of the horn can affect the loudspeaker behavior in an unpredictable way. Therefore, the usable bandwidth of this type of source is typically rather limited. For example, Speed et al. reported an upper band limit of 4 kHz, i.e., the frequency of the first marked zero that could not be equalized due to the limited dynamic range of the loudspeaker [13].

Similar to this is the use of an in-ear headphone as a glottal source [11]. However, to our knowledge, the effect of the vocal tract load on the acoustic excitation of such a headphone has not been examined yet. Another method to produce a well-defined glottal volume velocity is based on a calibrated impedance head connected to the glottal end of the model [16]. This technique allows high precision and dynamic range over a wide frequency range, but requires a sophisticated calibrated impedance head with three measurement microphones.

**Fig 1:**
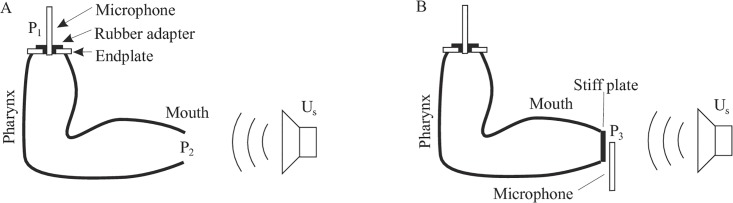
Experimental setup for measuring the vocal tract transfer function. (A) The mouth of the vocal tract model is open and the pressure *P*_1_ is measured at the glottis. (B) The mouth of the model is closed and the pressure *P*_3_ is measured right in front of the closed mouth.

The other general approach to measure the transfer function is to excite the vocal tract model with an external sound source *P*_s_(*ω*) in front of the lips and measure the sound pressure *P*_1_(*ω*) inside the model at the glottal end [1, 2, 10] as illustrated in Fig 1A. This method avoids the intricate problem of constructing a suitable volume velocity source and only requires an ordinary wideband loudspeaker and a microphone. Kitamura et al. [10] argued that

$$\begin{array}{c} P_{1}\left(\omega \right)=\left[1+Z_{r}\left(\omega \right)/Z_{0}\right]\cdot P_{s}\left(\omega \right)\cdot H\left(\omega \right), \end{array}$$ (1)

where *H*(*ω*) = *U*_2_(*ω*)/*U*_1_(*ω*) is the volume velocity transfer function between the volume velocities *U*_1_(*ω*) through the glottis and *U*_2_(*ω*) through the lips (see Eqs A⋅4 and A⋅10 in Kitamura et al. [10]), which is usually used to characterize vocal tract acoustics, *Z*_r_(*ω*) is the radiation impedance, and *Z*_0_ is the characteristic impedance of a plane wave. Therefore, if the frequency response *P*_s_(*ω*) of the source is assumed to be independent of frequency, *P*_1_(*ω*) is close to *H*(*ω*) in terms of formant frequencies, but the spectral tilt is different because *Z*_r_ is monotonically increasing with frequency. So far, the magnitude of this tilt and hence the deviation of *P*_1_ from the true volume velocity transfer function has not been examined. In principle, the spectral tilt could be compensated by an adapted source. However, this presupposes the exact knowledge of the source characteristics, i.e., one must be able to quantify the behavior of the source coupled with the vocal tract models. Since in many cases the sources are not independent of the model, this compensation would have to be explicitly determined for each configuration. According to Eq (1), it seems likely that the deviation of *P*_1_(*ω*) from *H*(*ω*) depends on the model geometry, because the radiation impedance *Z*_r_(*ω*) depends on the mouth aperture and the shape of the lips.

The purpose of this paper is to examine the extent to which *P*_1_ differs from the true volume velocity transfer function for different vocal tract shapes and to propose an extension to Kitamura’s method that allows the *precise* determination of the volume velocity transfer function. Therefore, we present an alternative description of the measurement situation in terms of an acoustic circuit model. The analysis of this circuit model shows that the precise volume velocity transfer function can be obtained with an additional sound pressure measurement in front of the closed lips of the vocal tract model (without the need for an actual acoustic flow measurement). This method is used to measure the volume velocity transfer functions of four physical vocal tract models and the results are compared to finite element simulations of the same models.

We must emphasize that the proposed method cannot be used to directly measure the volume velocity transfer function of the real vocal tract *in vivo*. Instead, the vocal tract performing the articulation of interest has to be scanned in an MRI scanner, and the vocal tract shape has to be segmented from the MRI data and printed as a 3D object. Despite this limitation, there are a range of applications for the proposed method. On the one hand, certain articulatory strategies during the production of phones in speech and singing can be precisely associated with changes in the acoustic transfer function. This may help to examine how professional singers tune the acoustic properties of their vocal tract when they sing at different pitches [11], or how we adapt vocal tract acoustics for different voice qualities (e.g., between spoken and shouted speech). On the other hand, the detailed and precise volume velocity transfer functions for realistic 3D vocal tract shapes can serve as ground truth for the validation of methods to transform 2D or 3D vocal tract models to plane-wave acoustic tube models in articulatory speech synthesis (e.g., [17, 18]). The transformation from 3D vocal tract models to low-dimensional acoustic tube models is necessary because full 3D acoustic simulations are far too slow for articulatory speech synthesis in real-time.

## Materials and methods

### Theory

In this section we show that the volume velocity transfer function of the vocal tract, i.e., the ratio of the volume velocity *U*_2_(*ω*) through the lips to the volume velocity *U*_1_(*ω*) through the glottis, corresponds exactly to a ratio of two pressures *P*_1_(*ω*) and *P*_3_(*ω*), which can be easily measured when the vocal tract is externally excited with a volume velocity source *U*_s_(*ω*) as in Fig 1A and 1B.

The pressure *P*_1_ is measured at the glottis while the mouth is open and the pressure *P*_3_ is measured right in front of the *closed* lips.

**Fig 2:**
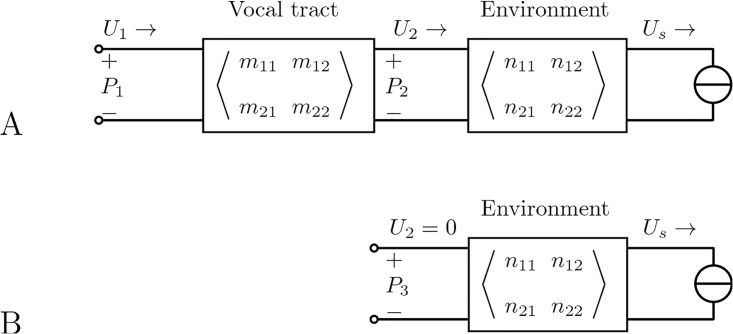
Theoretical model of the measurement setup. Equivalent acoustic circuits for the measurement situations in Fig 1A and 1B, respectively.

We start by modeling the measurement situation in Fig 1A with the general acoustic circuit in Fig 2A.

Here, the vocal tract is represented in terms of a two-port network where the input (= glottal) pressure *P*_1_ and the input volume velocity *U*_1_ are related to the output pressure *P*_2_ and the output volume velocity *U*_2_ by a 2 × 2 transmission matrix *M*(*ω*) = [*m*_*ij*_(*ω*)] as follows:

$$\begin{array}{c} \left(\begin{array}{c} P_{1}\\ U_{1} \end{array})=(\begin{array}{cc} m_{11} & m_{12}\\ m_{21} & m_{22} \end{array})\cdot (\begin{array}{c} P_{2}\\ U_{2} \end{array}\right) \end{array}$$ (2)

The transmission of sound between the mouth opening and the location of the external sound source is correspondingly modeled by a two-port network with the transmission matrix *N*(*ω*) = [*n*_*ij*_(*ω*)]. Furthermore, let *O* = [*o*_*ij*_(*ω*)] = *M* ⋅ *N* denote the joint transfer matrix between the glottis and the external source. Due to the principle of reciprocity [19], det *M* = 1, det *N* = 1 and det *O* = 1. Considering that *U*_1_ = 0, the sound pressure *P*_1_ measured at the glottis can be expressed as a function of volume velocity *U*_s_ at the sound source:

$$\begin{array}{c} P_{1}=(o_{12}-\frac{o_{11}o_{22}}{o_{21}})U_{s}. \end{array}$$ (3)

We now consider the case where the mouth of the vocal tract is closed with a plate and the sound source is at the same position as before, as shown in Fig 1B. In this case, the volume velocity *U*_2_ through the mouth of the model is zero. The pressure *P*_3_ that is measured in front of the closed lips is now an open-circuit pressure, as shown by the equivalent circuit in Fig 2B. It can be expressed as a function of the volume velocity *U*_s_ at the sound source as follows:

$$\begin{array}{c} P_{3}=(n_{12}-\frac{n_{11}n_{22}}{n_{21}})U_{s}. \end{array}$$ (4)

From Eqs (3) and (4) we can form the ratio *P*_1_/*P*_3_:

$$\begin{array}{c} \frac{P_{1}}{P_{3}}=\frac{n_{21}\left(o_{11}o_{22}-o_{12}o_{21}\right)}{o_{21}\left(n_{11}n_{22}-n_{12}n_{21}\right)}=\frac{1}{m_{21}\left(n_{11}/n_{21}\right)+m_{22}} \end{array}$$ (5)

The quotient *n*_11_/*n*_21_ on the right-hand side of Eq (5) equals the input impedance *P*_2_/*U*_2_ of the two-port network for the “environment” in Fig 2, which is equivalent to the radiation impedance *Z*_r_ of the vocal tract. This can be proven by considering the two-port network described by the transmission matrix *N*(*ω*), which represents the exterior space between the vocal tract and the external sound source (loudspeaker). The equations to describe the transfer characteristics are as follows:

$$\begin{array}{ccc} P_{2} & = & n_{11}P_{s}+n_{12}U_{s} \end{array}$$ (6)

$$\begin{array}{ccc} U_{2} & = & n_{21}P_{s}+n_{22}U_{s}. \end{array}$$ (7)

For the case of an inactive loudspeaker, one gets *U*_*s*_ = 0, and the ratio

$$\begin{array}{c} \frac{P_{2}}{U_{2}}=\frac{n_{11}}{n_{21}}=Z_{r} \end{array}$$ (8)

can be derived. It should be noted that this equation is only an approximation that presumes that the presence of the loudspeaker does not essentially change the exterior space. This assumption holds the smaller the loudspeaker and the greater the distance from the loudspeaker to the resonator.

Combining Eqs (5) and (8), we obtain

$$\begin{array}{c} \frac{P_{1}}{P_{3}}=\frac{1}{m_{21}Z_{r}+m_{22}}. \end{array}$$ (9)

This pressure ratio is exactly the desired volume velocity transfer function *H* = *U*_2_/*U*_1_ of the vocal tract, which is easily verified with the second equation in (2) by setting *P*_2_ = *U*_2_*Z*_r_.

Note that for both cases shown if Fig 2A and 2B, *U*_s_ is assumed to be unaffected by the configuration (open or closed mouth) of the vocal tract model under test, because the whole vocal tract model constitutes a small reflecting surface at a certain distance from the loudspeaker in an otherwise open acoustic space. It is also worth noting that the measurement situation depicted in Fig 1A closely resembles the hearing situation when a sound wave emitted from a volume source *U*_s_ hits the human ear. In this analogy, the vocal tract corresponds to the ear canal, and its glottal end corresponds to the eardrum. The hearing situation has been well studied in the context of binaural hearing [20] and can be adapted to obtain the same results as we did above.

### Preparation of the physical vocal tract models

To test the theory above, four physical vocal tract models were created. Three of the models represent the vocal tract shapes for the vowels /a/, /u/ and /i/ produced by a 24-year-old male native German subject. The participant was a singing student at the Voice Research Laboratory, Hochschule für Musik Carl Maria von Weber Dresden, with whom one of the co-authors cooperates. The MRI images were taken in 07/2015. Data aquisition within this study was approved by the ethical review committee of the medical faculty “Carl Gustav Carus” of the TU Dresden (EK153042011). After having been informed about risk and procedures, the participant provided written consent. The shapes were obtained from MRI data of the vocal tract according to a procedure presented in detail before [21, 22]. In brief, the subject produced each of the two vowels for about 12.1 s, while his vocal tract was scanned using a 3 T MRI machine (Magnetom Trio Trim, Siemens Medical Solutions, Erlangen, Germany). The MRI was performed with a 12-element-head-neck-coil using a 3D volume-interpolated-breathhold-examination sequence with 1.22 ms/4.01 ms (echo time/repetition time), flip angle 9°, a field-of-view of 300 × 300 mm^2^, a matrix of 288 × 288 pixels^2^, 52 sagittal slices and a slice thickness of 1.8 mm.

The vocal tract cavities in the MRI data were segmented using IPTools [23], slightly smoothed, and exported as triangle meshes representing the vocal tract walls. The termination of the vocal tract models at the lips was approximated by a plane parallel to the coronal plane. The anteroposterior position of this plane was set to the place where the vertical distance between the midsagittal contours of the upper and lower lips reached its minimum. The lateral gaps of the vocal tract in the region of the lips between the corners of the mouth and the termination plane were manually closed during the segmentation.

Because the teeth are invisible in MRI, they were separately reconstructed as triangle meshes by scanning plaster models of the subject’s mandibular and maxilla using a NextEngine desktop 3D laser scanner. All triangle meshes were then voxelized with a voxel size of 0.25 × 0.25 × 0.25 mm^3^ using binvox [24] (www.patrickmin.com/binvox/) and merged into a single voxel model using 3DSlicer [25] (www.slicer.org). The teeth were positioned relative to the vocal tract cavities by careful visual inspection. The merged voxel models for /a/, /u/ and /i/ were then converted back into triangle meshes using 3DSlicer. The free software package Meshlab (www.meshlab.sourceforge.net) was then used for adaptive mesh simplification and extrusion of the surfaces to create vocal tract walls with a thickness of 3 mm. Finally, the programs netfabb Basic (www.netfabb.com) and ParaView (www.paraview.org) were used to repair defects in the triangle meshes and separate the models into two halves suitable for 3D printing. The model halves were printed with a 3D printer (ULTIMAKER 2, www.ultimaker.com) using polylactic acid (PLA) material, and the two halves of each /a/, /u/ and /i/ were conglutinated (S1 File). In addition to the realistic models for /a/, /u/ and /i/, a uniform tube of 170 mm length and 27.6 mm inner diameter was created as a fourth model (denoted as /Ə/ in the following) and printed in one piece with the 3D printer.

**Fig 3:**
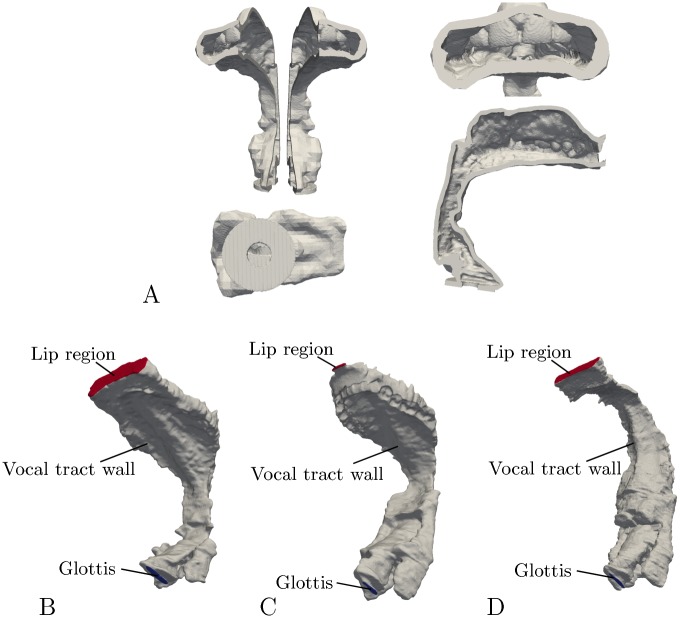
Printed and finite element models. (A) Different views of the 3D-printed model of /a/. (B), (C) and (D) Finite element models of /a/, /u/ and /i/. For each model, the surface was partitioned into three regions representing the glottis, the lip region and the vocal tract walls.

All four models were terminated at the glottal end with a uniform endplate of 3 mm thickness and a hole with 10 mm diameter to allow inserting the measurement microphone with a rubber adapter (Figs 1A and 3A).

Finally, the printed models were covered with plaster of a thickness of about 1 cm to increase the mass of the walls and avoid sound radiation from the model surfaces.

### Measurement of the transfer functions of the physical models

For each of the four physical models, the volume velocity transfer function was obtained according to the theory outlined in Section II.A by two successive sound pressure measurements (*P*_1_ and *P*_3_) with the setups in Fig 1A and 1B. During both measurements, the model was excited by an external sound source *U*_s_ (VISATON speaker, type FR 10-8 Ohm in a custom-made cylindrical enclosure) producing an exponential sweep with a power band from 100–10000 Hz and a duration of 21 s according to the method by Farina [26]. The sound source was located 25 cm in front of the mouth opening to prevent near-field effects on the model. The pressure *P*_1_ was recorded with a 1/4” measurement microphone (type MK301E/MV310, www.microtechgefell.de) inserted into the glottal end of the model so that the microphone membrane was flush with the upper surface of the “vocal folds”. For the measurement of *P*_3_, the mouth opening of the model was closed with a stiff plate of 3 mm thickness and the size of the mouth, fixed to the model with two-sided tape. *P*_3_ was measured right in front of this plate using a probe microphone (ER-7C, www.etymotic.com). For each model, the transfer function was calculated as *H*(*ω*) = *P*_1_(*ω*)/*P*_3_(*ω*). In addition to *P*_1_ and *P*_3_, the free-field sound pressure *P*_ref_(*ω*) produced by the loudspeaker was measured in the absence of the model at the position where the mouth was. All measurements were conducted in an anechoic chamber at a room temperature of 20° C.

### Calculation of the transfer functions with finite element models

For comparison with the physical measurements above, the volume velocity transfer functions of the four vocal tract models were calculated using the finite element method (FEM). The FE model creation and the numerical simulation were performed similarly to Fleischer et al. [22]. Accordingly, the volume meshes (S2 File) of the four vocal tract shapes in Sec. II.B were created from the surface representations using the software Gmsh [27]. The mesh for the vowel /a/ had a mean element size of 1.91 mm and 101,240 degrees of freedom (DOF), the mesh for /u/ had a mean element size of 2.19 mm and 78,568 DOF, the mesh for /i/ had a mean element size of 1.68 mm and 124,650 DOF, and for /Ə/, the mesh had 31,048 DOF and an element size of 3.01 mm. Note, that the geometrically simple /Ə/ has a slightly greater element size, because of they are no tiny details. In order to validate the numerical results, the polynomial degree of the shape functions was varied (for 2nd order polynomials the DOF increased to about 230,000 for /Ə/ and up to 900,000 for /i/). The comparison of the simulation results for first and second order polynomials showed that the chosen element size was sufficient for all finite element models in the investigated frequency range even with linear shape functions.

The acoustic simulation was performed with the open-source software FEniCS (http://fenicsproject.org; [28]) based on the Helmholtz equation

$$\begin{array}{c} -(\kappa^{2}+\nabla^{2})P\left(\vec{x},\omega \right)=0, \end{array}$$ (10)

where *P* is the complex-valued sound pressure as a function of the position $\vec{x}$ and the angular frequency *ω*, *κ* = *ω*/*c* is the wave number, and *c* = 343 m/s is the speed of sound for a temperature of 20° Celsius. The particle velocity $V(\vec{x},\omega )$ is related to the sound pressure by $\nabla P=-j\omega \varrho \vec{V}$, where *ϱ* = 1.20 kg/m^3^ is the ambient density for a temperature of 20° Celsius. For the computation of the volume velocity transfer function, the following boundary conditions were applied:

$$\begin{array}{ccc} \nabla P_{\text{glottis}}\cdot \vec{n} & = & -j\omega \varrho V_{0}\\ \nabla P_{\text{wall}}\cdot \vec{n} & = & -j\kappa \frac{\varrho c}{Z_{\text{wall}}}P_{\text{wall}}\\ \nabla P_{\text{lips}}\cdot \vec{n} & = & -j\kappa \frac{\varrho c}{Z_{r}}P_{\text{lips}}. \end{array}$$ (11)

Here, *P*_glottis_ is the pressure on the model surface region representing the glottis, *P*_lips_ is the pressure on the surface region representing the lip opening, and *P*_wall_ is the pressure on the surface of the vocal tract walls (see Fig 3B and 3C for the individual regions). Furthermore, $\vec{n}$ is the outward normal vector of the mesh surface and the wall impedance *Z*_wall_ was empirically set to 500·*ϱc* = 205,800 kg/(m⋅s)^2^ for appropriate damping. Since the wall impedances of the printed 3D models are not known, the simplest model (/Ə/) was used to adjust the wall impedance in such a way that the transfer function was well approximated but did not change too much in comparison to the solution for the hard-walled model. The background is that for this simple model—due to small reflections within the model—the wall damping must have a small influence. The estimated value was then adopted for the other models. It is conceivable that the wall impedance depends on location and frequency (see [22]), but in order to limit the calculation effort, a constant value was used. The radiation impedance *Z*_r_ was set to that of a rigid piston with a radius $r_{\text{lips}}=\sqrt{A_{\text{lips}}/\pi }$ acting into an infinite baffle [22, 29]. The acoustic pressure *P*_*lips*_ at the lip opening was determined in the center of the area representing the lip region. Based on these boundary conditions, the transfer function *H*_FEM_(*ω*) = *U*_lips_(*ω*)/*U*_glottis_(*ω*) was calculated, where *U*_lips_ = *A*_lips_ ⋅ *V*_lips_, *U*_glottis_ = *A*_glottis_ ⋅ *V*_glottis_, and *A*_lips_ and *A*_glottis_ are the cross-sectional areas of the lips and the glottis, respectively. For each of the models, the transfer function was calculated with a frequency resolution of 3 Hz from 0 to 6 kHz, taking up to 8 h time per model on a standard desktop computer.

## Results and discussion

**Fig 4:**
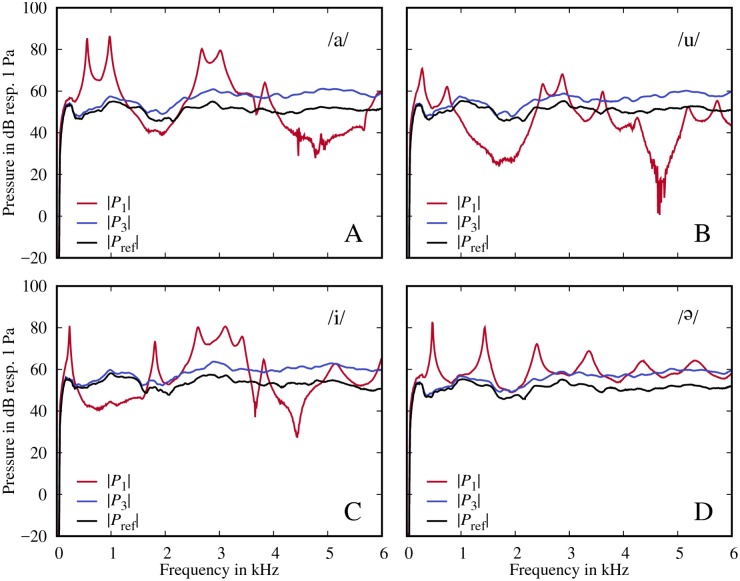
Measured signals. Spectra of pressure signals measured at the glottis (*P*_1_), in front of the closed mouth (*P*_3_) and without the models (*P*_ref_) for the models /a/ (A), /u/ (B), /i/ (C) and /Ə/ (D).

Fig 4A–4D show the pressure *P*_1_ measured at the glottis, the pressure *P*_3_ measured right in front of the closed lips, and *P*_ref_ measured without the model in front of the loudspeaker for each /a/, /u/, /i/ and /Ə/.

**Fig 5:**
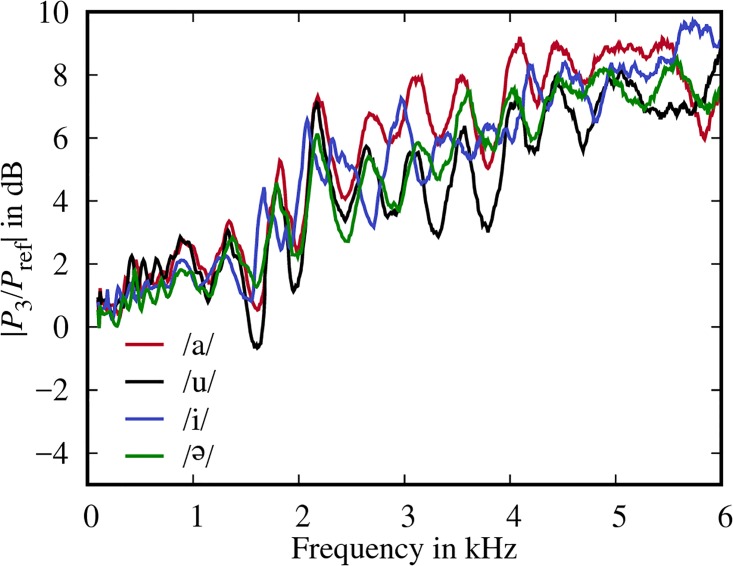
Experimentally determined pressure ratios at the lips. Ratio of the pressure spectra measured in front of the closed mouth (*P*_3_) and without the models (*P*_ref_) for all four models.

Here, it can be seen that the *P*_1_ spectra resemble typical volume velocity transfer functions for these vowels as claimed by Kitamura et al. [10]. However, we also see a clear drift between the spectra for *P*_3_ and *P*_ref_ with differences of up to 10 dB at 6 kHz. Fig 5 shows that the drifts are generally similar for all four models, but that they differ in detail.

The drift differences between the models are smaller than we initially expected, because the radiation impedance *Z*_r_ in Eq (1) suggests that the drift depends on the mouth aperture (which varies from 0.44 cm^2^ for /u/ to 5.98 cm^2^ for /Ə/ in our models). On the other hand, the similarity is less surprising in the context of the actual measurement setup, because *P*_3_ was measured in front of the *closed* mouths of the models.

**Fig 6:**
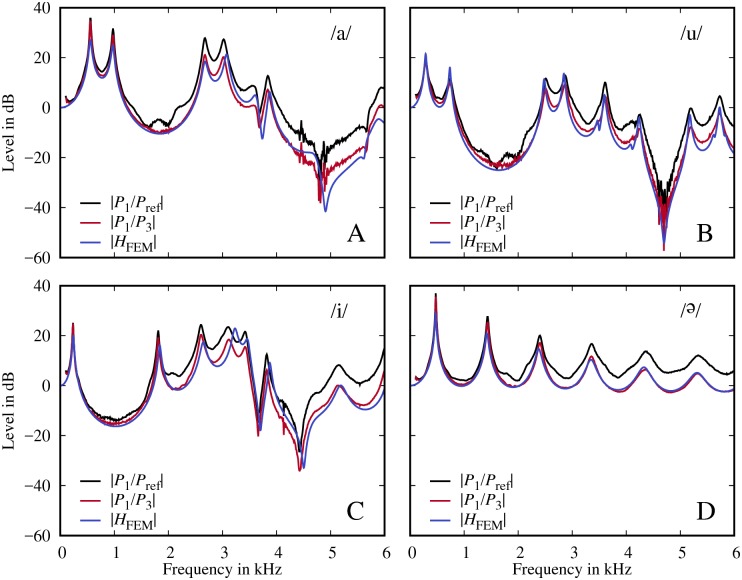
Calculated measures. Transfer functions *P*_1_/*P*_3_, *P*_1_/*P*_ref_, and the simulated transfer functions *H*_FEM_ for the models /a/ (A), /u/ (B), /i/ (C) and /Ə/ (D).

Fig 6A–6D show the ratios of the pressure spectra *P*_1_/*P*_3_ (which is theoretically equivalent to the volume velocity transfer function) and *P*_1_/*P*_ref_ (which just compensates for the frequency response of the loudspeaker, as was done by Delvaux & Howard [2], for example), as well as the volume velocity transfer functions *H*_FEM_ calculated with the FEM.

The spectra *P*_1_/*P*_3_ and *P*_1_/*P*_ref_ clearly reflect the difference between *P*_3_ and *P*_ref_ in Fig 4A–4D. It is also notable that the proposed pressure ratio *P*_1_/*P*_3_ is much closer to the FE calculation than *P*_1_/*P*_ref_ for all four models. The RMS spectral differences in the 0 − 6 kHz range between *P*_1_/*P*_3_ and *H*_FEM_ are 3.6 dB, 2.8 dB, 3.8 dB and 1.0 dB for the models /a/, /u/, /i/ and /Ə/, respectively, while they are as high as 7.8 dB, 7.0 dB, 7.3 dB and 5.4 dB between *P*_1_/*P*_ref_ and *H*_FEM_. Notably, the usage of *P*_*ref*_ as the denominator in the volume velocity transfer functions has some limits, despite the fact that *P*_*ref*_ is supposed to correct for the loudspeaker spectral characteristics. If one considered Fig 2B and assumed that the voval tract model was not there (*U*_2_ ≠ 0) the leading equations would be

$$\begin{array}{c} P_{ref}=n_{11}P_{s}+n_{12}U_{s} \end{array}$$ (12)

$$\begin{array}{c} U_{2}=n_{21}P_{s}+n_{22}U_{s}. \end{array}$$ (13)

After some analysis, and the relation *Z*_*r*_ = *n*_11_/*n*_21_ shown above, one gets

$$\begin{array}{c} \frac{P_{1}}{P_{ref}}=\frac{1}{m_{21}Z_{r}+m_{22}}\cdot \frac{U_{s}}{U_{s}-n_{11}U_{2}}. \end{array}$$ (14)

Comparing Eq (14) with Eq (9), one can see at least two significant differences. First, the ratio *P*_1_/*P*_*ref*_ depends on *U*_*s*_ which in turn is not valid as we are interested in a transfer function which is, by definition, not dependent on the excitation. Secondly, this serious problem can only be bypassed if either *n*_11_ = 0 (this is in conflict with the principle of reciprocity) or *U*_2_ = 0 (lips closed). Both options are not valid. Further, implementation of an arbitrary shaped stiff plate to force *U*_2_ to be zero is also not a valid approach, because for this case the transmission matrix *N*(*ω*) would by changed significantly, which in turn would affect the subsequent analysis.

However, calculating the proposed pressure ratio *P*_1_/*P*_3_ not only prevents the general drift compared to the true volume velocity transfer function, but may also prevent spurious spectral “defects”, which might be misinterpreted as true spectral information. For example, the spurious peaks in *P*_1_/*P*_ref_ at around 2 kHz disappear in *P*_1_/*P*_3_ due to the normalization by *P*_3_ (see Fig 6A–6D).

A notable feature for /a/, /i/ and /u/ (Fig 6A–6C)—in contrast to /Ə/ (Fig 6D)—is that there are strong zeros between 4-5 kHz. These zeros are known to be caused by the sinus piriformes which are side cavities of the main vocal tract [2]. These side cavities are not present in /Ə/.

To assess a potential effect of the measurement method on the formant frequencies, the first four formant frequencies were determined by peak picking in the magnitude spectra of *P*_1_/*P*_3_, *P*_1_/*P*_ref_ and *H*_FEM_ for all four models. The results are given in Table 1, together with the relative formant deviations between the formants in *P*_1_/*P*_3_ compared to *H*_FEM_ and *P*_1_/*P*_ref_ compared to *H*_FEM_. The average formant deviation between *P*_1_/*P*_3_ and *H*_FEM_ is 1.074%, and the average formant deviation between *P*_1_/*P*_ref_ and *H*_FEM_ is 1.131%. Hence, the formants of the two measured transfer functions are similarly equal to the formants of the reference FE simulation. The overall deviation between measured and simulated formants is less than 2%, which is much smaller than the differences reported in the few previous studies that made similar comparisons [1, 13].

**Table 1:** Formant frequencies in Hz of the simulated and measured transfer functions and their relative deviations in %.

	/a/				/u/				/i/				/Ə/			
	*F*_1_	*F*_2_	*F*_3_	*F*_4_	*F*_1_	*F*_2_	*F*_3_	*F*_4_	*F*_1_	*F*_2_	*F*_3_	*F*_4_	*F*_1_	*F*_2_	*F*_3_	*F*_4_
*H*_FEM_	557	971	2680	3077	288	737	2481	2846	241	1841	2646	3233	472	1420	2379	3348
*P*_1_/*P*_3_	557	975	2676	3020	289	740	2510	2869	235	1812	2606	3121	476	1434	2402	3354
*P*_1_/*P*_ref_	558	975	2675	3024	289	741	2513	2869	235	1812	2604	3106	476	1434	2400	3358
100% ⋅ (*F*_*i*_(*P*_1_/*P*_3_)/*F*_*i*_(*H*_FEM_) − 1)	0.00	0.41	-0.15	-1.85	0.35	0.41	1.17	0.81	-2.49	-1.58	-1.51	-3.46	0.85	0.99	0.97	0.18
100% ⋅ (*F*_*i*_(*P*_1_/*P*_ref_)/*F*_*i*_(*H*_FEM_) − 1)	0.18	0.41	-0.19	-1.72	0.35	0.54	1.29	0.81	-2.49	-1.58	-1.59	-3.93	0.85	0.99	0.88	0.30

In addition, Tables 2 & 3 show the bandwidths and amplitudes of the resonances of all models and their deviation from the finite element models. The average bandwidth and amplitude deviation between *P*_1_/*P*_3_ and *H*_*FEM*_ are 25.3 Hz and 4.1 dB, and between *P*_1_/*P*_*ref*_ and *H*_*FEM*_ these values are 31.6 Hz and 5.1 dB. Also here, there are no big differences between the two measured volume velocity transfer functions. It should be kept in mind that these values strongly depend on the selected wall impedance *Z*_*wall*_. It is quite possible to optimize the values in such a way that the deviations of amplitudes and bandwidths are minimized. However, this would go beyond the scope of this work.

**Table 2:** Bandwidths in Hz of the simulated and measured transfer functions and their absolute deviations in Hz.

	/a/				/u/				/i/				/Ə/			
	*BW*_1_	*BW*_2_	*BW*_3_	*BW*_4_	*BW*_1_	*BW*_2_	*BW*_3_	*BW*_4_	*BW*_1_	*BW*_2_	*BW*_3_	*BW*_4_	*BW*_1_	*BW*_2_	*BW*_3_	*BW*_4_
*H*_FEM_	43.3	44.9	86.1	72.6	30.1	30.0	40.2	39.2	25.4	41.9	97.5	93.7	21.12	52.2	104.0	166.1
*P*_1_/*P*_3_	18.4	30.6	69.9	92.3	48.6	80.5	80.0	84.4	14.2	29.9	78.8	140.3	10.1	33.1	79.4	133.7
*P*_1_/*P*_ref_	18.6	30.7	67.9	88.5	47.8	87.9	85.5	84.1	14.2	29.6	78.3	163.5	10.1	32.9	79.5	135.0
|*BW*_*i*_(*P*_1_/*P*_3_) − *BW*_*i*_(*H*_FEM_)|	24.9	14.3	16.1	19.7	18.5	50.5	39.8	45.2	11.2	12.0	18.8	46.6	11.0	19.1	24.6	32.4
|*BW*_*i*_(*P*_1_/*P*_ref_) − *BW*_*i*_(*H*_FEM_)|	24.7	14.1	18.1	15.8	17.6	57.9	45.3	44.9	11.2	12.3	19.2	69.8	11.0	19.3	24.5	31.1

**Table 3:** Amplitudes in dB of the simulated and measured transfer functions and their absolute deviations in dB.

	/a/				/u/				/i/				/Ə/			
	*A*_1_	*A*_2_	*A*_3_	*A*_4_	*A*_1_	*A*_2_	*A*_3_	*A*_4_	*A*_1_	*A*_2_	*A*_3_	*A*_4_	*A*_1_	*A*_2_	*A*_3_	*A*_4_
*H*_FEM_	27.0	25.0	18.5	21.3	21.5	16.1	11.4	13.6	20.0	16.2	17.4	22.9	29.0	21.0	14.7	10.4
*P*_1_/*P*_3_	35.4	29.1	21.1	20.2	18.5	9.8	7.7	9.3	25.5	19.4	20.4	18.5	36.4	25.6	17.1	11.7
*P*_1_/*P*_ref_	36.7	31.7	27.9	27.4	19.3	11.4	11.7	12.9	25.7	22.1	24.4	23.5	37.8	27.9	20.1	16.7
|*A*_*i*_(*P*_1_/*P*_3_) − *A*_*i*_(*H*_FEM_)|	8.3	4.2	2.7	1.1	3.0	6.2	3.7	4.3	5.5	3.2	3.0	4.4	7.4	4.6	2.4	1.3
|*A*_*i*_(*P*_1_/*P*_ref_) − *A*_*i*_(*H*_FEM_)|	9.6	6.7	9.4	6.0	2.2	4.6	0.3	0.7	5.7	5.9	7.0	0.5	8.7	6.9	5.4	6.4

A limitation of this study is the approximation of the lip openings of the vocal tract models in terms of straight cuts. For most speech sounds, the lips form a wedge-like opening of the vocal tract, which have non-negligible acoustic effects [12]. When the proposed method is used to measure the volume velocity transfer functions of vocal tract models with such realistic lip shapes, the precise positioning of the microphone in front of the closed lips might play a role, and closing the mouth may become more complicated (modeling clay could be used). Furthermore, it might become necessary to measure the pressure at multiple points on the outer double-curved surface of the closed lips. It can be expected that all these individual transfer functions differ slightly from each other. The averaged transfer function should then be considered as the result. However, in most cases we would expect that the averaged transfer function is very close to the one that is obtained when *P*_3_ is measured in the midsagittal plane in the middle between the upper and lower lip. This issue deserves further investigation.

## Conclusion

In this paper we presented a precise method for the measurement of the volume velocity transfer function of 3D-printed models of the vocal tract based on acoustic excitation with an external sound source, which avoids the obstacles and limitations involved in transfer function measurements with a glottal source, requires little special equipment (except the necessity of an anechoic chamber), and is simple to conduct. This method is an extension of the approach presented by Kitamura et al. [10] and has the advantage that the relative levels of the measured resonance peaks correspond to those of the true volume velocity transfer function, and that the *overall* level of the transfer function corresponds to the true level (i.e., a level of 0 dB at a frequency of 0 Hz). Furthermore, we have investigated the resulting deviation that happens without the proposed normalization. This deviation consists of a general upward drift of the spectral level with increasing frequency, and is relatively independent from the vocal tract model geometry. However, the fine structure of the spectral drift may introduce spurious peaks or troughs into the transfer function, which may cause misinterpretations. The proposed technique prevents this problem and facilitates a more accurate acoustic characterization of the increasingly used 3D-printed vocal tract models in speech and singing research than before. Although the presented procedure is not applicable to *in vivo* situations, it has a range of applications in basic phonetic research and the potential to improve methods for articulatory speech synthesis. Finally, the proposed method is not limited to models of the vocal tract but can be used for most kinds of tube-like acoustic resonators.

## Supporting information

S1 File Printing models. Files containing the printing models of the vowels /a/, /u/, /i/, and /Ə/.
(ZIP)

S2 File Volume meshes of the finite element models. Files containing the volume meshes as used for finite element modeling of the vowels /a/, /u/, /i/, and /Ə/.
(ZIP)
